# ﻿*Aspidistradaibuensis* var. *longkiauensis*, a new variety of *Aspidistra* (Asparagaceae) from Taiwan, identified through morphological and genetic analyses

**DOI:** 10.3897/phytokeys.222.100885

**Published:** 2023-03-22

**Authors:** Chang-Tse Lu, Ming-Jen Yang, Min-Xin Luo, Jenn-Che Wang

**Affiliations:** 1 Department of Biological Resources, National Chiayi University, 300 Syuefu Rd., Chiayi City 60004, Taiwan National Chiayi University Chiayi Taiwan; 2 Department of Life Science, National Taiwan Normal University, 88 Ting-Chow Rd., Sec 4, Wenshan, Taipei City 11677, Taiwan National Taiwan Normal University Taipei Taiwan

**Keywords:** *Aspidistra*, microsatellite, morphometric analysis, new variety, Taiwan

## Abstract

*Aspidistra* Ker Gawl. is one of the the most diverse and fastest-growing genera of angiosperm. Most *Aspidistra* species have been discovered in a limited area or a single site through morphological comparison. Because of the lack of population studies, morphological variation within species and the boundaries of some species remain unclear. In recent years, combining genetic and morphological markers has become a powerful approach for species delimitation. In this study, we performed population sampling and integrated morphometrics and microsatellite genetic diversity analyses to determine the species diversity of *Aspidistra* in Taiwan. We identified three species, namely *Aspidistraattenuata* Hayata; A.daibuensisHayatavar.daibuensis; A.mushaensisHayatavar.mushaensis; and reduced *A.longiconnectiva* C.T.Lu, K.C.Chuang & J.C.Wang to the variety level, and described a new variety, A.daibuensisHayatavar.longkiauensis. The description, diagnosis, distribution, and photographs of this new variety as well as a key to the known Taiwanese *Aspidistra* are provided.

## ﻿Introduction

The genus *Aspidistra* Ker Gawl. (Asparagaceae) is native to eastern and southeastern Asia, particularly China and Vietnam (Tillich 2014), and *Aspidistra* species typically grow under forest canopies and shrubs in high-rainfall areas. Plants of *Aspidistra* are characterized by a perennial herbaceous habit, conspicuous rhizomes, solitary or two to four tufts’ leaves, a variety of fruits and a highly diversified flower structure (Li et al. 2000; Lin et al. 2010). *Aspidistra* is one of the fastest growing genera in Angiosperm (Tillich and Averyanov 2018). In the past 15 years, the number of *Aspidistra* has considerably increased from approximately 93 taxa (Tillich 2008) to more than 200 species (Kalyuzhny et al. 2022). So far, new species of this genus have been discovered continuously. However, phylogenetic analysis of this genus has not yet been complete performed.

In Taiwan, Hayata (1912, 1920) described three species, namely *Aspidistraattenuata* Hayata, *A.daibuensis* Hayata, and *A.mushaensis* Hayata. However, the boundaries of some of these species are inconsistent. For example, Ying (2000) described that these three species should be combined into *A.elatior* Blume and regarded them as a variety, namely A.elatiorvar.attenuata (Hayata) Ying. Subsequently, Yang et al. (2001) demonstrated that the previously described three species differed from *A.elatior*, and that *A.mushaensis* is a synonym of *A.attenuata* Hayata. Wang (2004) and Chao (2016) have accepted the species concept of Hayata and reported that *A.daibuensis* is distributed in south and east Taiwan. However, Lu et al. (2020) reported that *A.daibuensis* is restricted to only south Taiwan and did not consider the population in east Taiwan. Wang (2004) suggested that *A.mushaensis* is distributed in not only central Taiwan but also south Taiwan. However, Lu et al. (2020) disagreed with this suggestion. A recent study suggested that the genus *Aspidistra* contains four species: *A.attenuata* Hayata; *A.daibuensis* Hayata; *A.longiconnectiva* C.T.Lu, K.C.Chuang, & J.C.Wang; and *A.mushaensis* Hayata (Lu et al. 2020).

Taxonomists have recognized nearly all *Aspidistra* species through the morphological comparison. However, this approach of species delimitation can cause some identification problems because of the intraspecific morphological variation or small morphological differences between closely related species (Pratt and Clark 2001; Whittall et al. 2004). Advances in many fields, such as molecular genetics, have helped taxonomists determine species boundaries and identify cryptic species (Whittall et al. 2004; Ellis et al. 2006; Bickford et al. 2007).

Population genetic methods, such as the algorithm developed by Pritchard et al. (2000) that was implemented in Structure software, can be used to identify well-differentiated genetic clusters and thus detect gene flow barriers. These methods can be employed to detect distinct species even if they are not yet reciprocally monophyletic at many genes due to incomplete lineage sorting (Duminil et al. 2015). In addition, by determining associations based on morphological data and considering the spatial distribution of detected genetic groups, we can investigate whether these genetic groups correspond to distinct species. Recently, many studies, such as those on *Ancistrocladus* Wall. (Turini et al. 2014), *Asteropyrum* J. R. Drumm. et Hutch. (Cheng et al. 2021), *Greenwayodendron* Verdc. (Lissambou et al. 2019), *Polygonum* L. (Mosaferi et al. 2016), and *Santiria* Blume (Ikabanga et al. 2017), have reported that the combination of morphological and population genetic data can be used to delineate species complexes.

In this study, we integrated morphological and genetic data to evaluate species delimitation within the genus *Aspidistra* in Taiwan. In addition, we conducted population genetic and morphometric analyses to confirm the previous classification hypotheses and to determine the number of species in the genus *Aspidistra* in Taiwan. We further performed genetic differentiation analysis for groups to determine whether they are the conspecific populations or distinct species.

## ﻿Materials and methods

### ﻿Recognition of *a priori* species groups

To classify collected samples into distinct morphological groups, we initially used a subjective approach in accordance with a previous taxonomic study (i.e., Lu et al. 2020). According to studies conducted by Wang (2004) and Chao (2016), the population in east Taiwan that was not considered in the study by Lu et al. (2020) was regarded as *A.daibuensis*. We defined distinct morphological groups as *a priori* groups and used them in this study.

We determined whether *a priori* groups correspond to distinct taxa based on the concept of iterative taxonomy (Yeates et al. 2011), and species hypotheses were iteratively assessed using classical morphometrics and microsatellite genetic data. First, we performed morphometric analyses, including multivariate and univariate analyses, to evaluate quantitative morphological characters. Subsequently, we inferred genetic groups from microsatellite markers by using Structure software and explored genetic diversity and differentiation between genetic groups. Finally, we compared the results of the two datasets and concluded species delimitation.

### ﻿Plant material sampling

**Figure 1.**
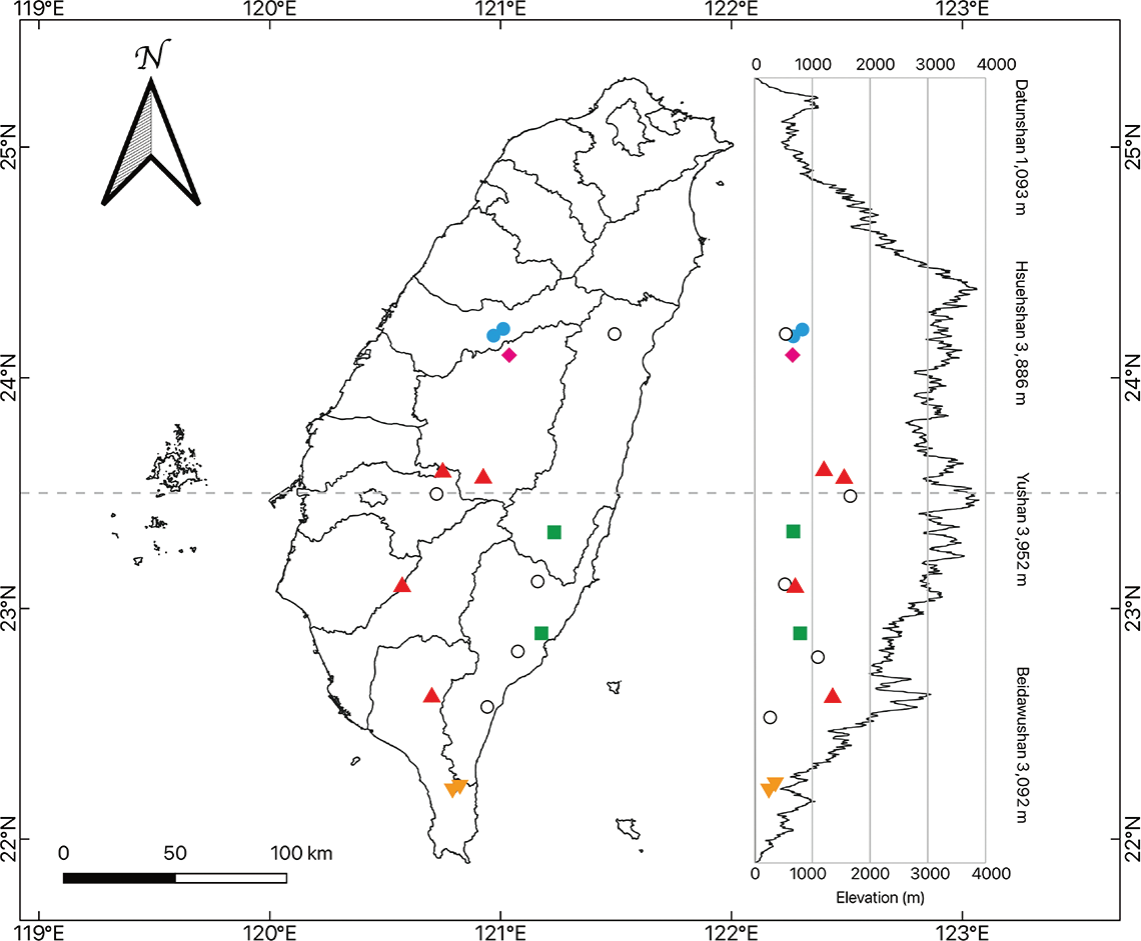
Sample collection location of the five *a priori Aspidistra* groups on the basis of morphological and geographical criteria. Color symbols: morphological and genetic data; white spots: only genetic data. Red triangle: AA, yellow inverted triangle: ADS, green square: ADE, fill diamond: AL, blue circle: AM.

For morphological comparison, living materials of *Aspidistra* were collected from Taiwan (Table 1, Fig. 1). A portion of these samples was converted into herbarium vouchers and deposited at TNU (The herbarium of National Taiwan Normal University, Taipei, Taiwan). For genotyping, the leaf material of these samples was dried using silica gel to preserve DNA and was stored at the Department of Life Sciences, National Taiwan Normal University, Taipei, Taiwan (Table 1, Fig. 1). In addition, herbarium specimens obtained from many herbaria (HAST, PPI, TAI, TAIF, and TNU; the acronyms are based on Index Herbariorum (https://sweetgum.nybg.org/science/ih/)) were examined directly, or digital plant specimens were evaluated online.

**Table 1.** Information on taxa, *a priori* groups, and locations at which samples were collected for morphological and genetic analyses.

Taxa	*A priori* group	Collected locations	Samples for morphometric analyses	Samples for DNA analyses	Abbrev.	Coordinates	Altitude (m)
*Aspidistramushaensis*	AM	Shaolai Trail, Taichung City	10	9	TS	24°12'21.59"N, 121°0'21.6"E	800
		Defulan Trail, Taichung City	12	12	TD	24°10'51.6"N, 120°58'33.59"E	600–700
*A.longiconnectiva*	AL	Huisun Experimental Forest Station, Nantou County	2	6	NHS	24°05'48.81"N, 121°02'13.99"E	600–701
*A.attenuata*	AA	Dongpu, Nantou County	11	11	N	23°33'51.81"N, 120°55'31.32"E	1500–1600
		Fengshan, Chiayi County	14	12	C	23°35'54.44"N, 120°44'45.7"E	1100–1300
		Dinghu, Chiayi County	0	8	H	23°29'27.16"N, 120°43'22.51"E	1600–1700
		Kantoushan Trail, Tainan City	2	4	T	24°10'39.97"N, 120°58'35.97"E	600–700
		Liuyishan Trail, Kaohsiung City	3	3	K	23°5'33.97"N, 120°34'26.97"E	600–800
		Peitawushan Trial, Pingtung County	16	16	P	22°36'52.88"N, 120°42'6.2"E	1200–1500
*A.daibuensis*	ADS	Shouka, Pingtung County	3	3	PK	22°14'34.79"N, 120°48'50.39"E	300–400
		Shuangliou, Pingtung County	11	10	PS	22°13'4.79"N, 120°47'38.39"E	200–300
		Jinlun, Taitung County	0	5	DJL	22°31'7.02"N, 120°54'40.10"E	100–300
		Lijia Forest Road, Taitung County	0	7	DLJ	22°48'57.80"N, 121°1'27.89"E	1000–1200
	ADE	Walami Trail, Hualien County	7	8	HW	23°19'40.21"N, 121°13'40.21"E	600–700
		Dulan Mountain Trail, Taitung County	6	6	DL	22°10'37.79"N, 121°10'37.79"E	700–900
		Wenshan Hot Spring , Hualien County	0	3	HWS	24°12'6.14"N, 121°29'26.61"E	600–700
		Lidao, Taitung County	0	5	NHL	23°8'30.60"N, 121°6'1.08"E	500–600
			97	128			

### ﻿Identification of morphological groups

**Figure 2.**
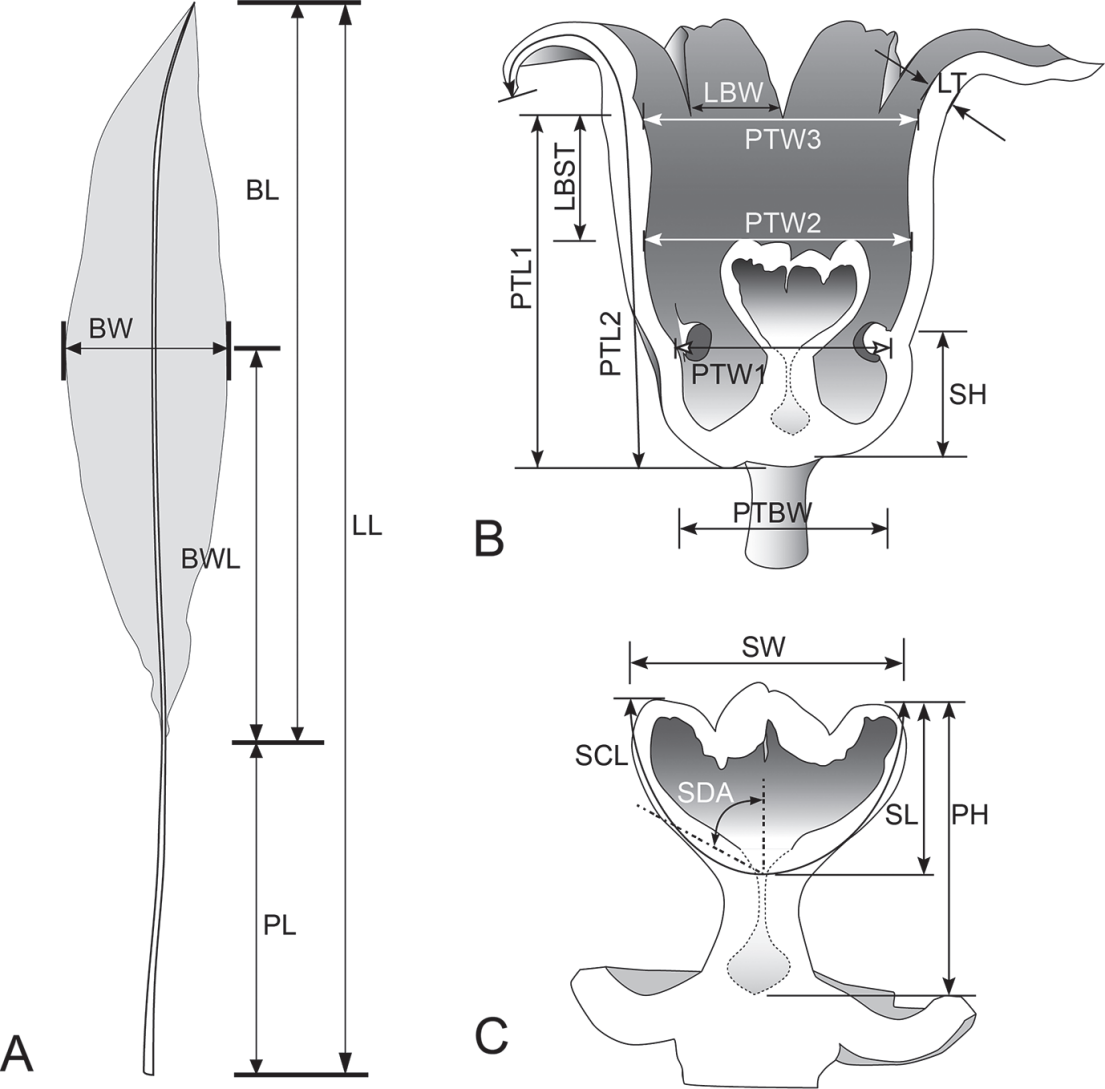
Morphological characters measured in this study **A** leaf characters **B, C** flower characters.

In the first step, we selected 128 fresh specimens representing different *a priori* groups, including available flowering specimens (N = 97; Table 1, Fig. 1). We used 5 leaf characters and 16 floral characters for 97 samples (Tables 2, 3, Fig. 2). Raw data were normalized before analysis. Subsequently, we used a clustering method (clustering analysis [CA]) and an ordination method (discriminant analysis [DA]) to project and visualize trends for morphological variability across our samples, including leaf and floral characters. Finally, we determined whether the mean of quantitative traits significantly differed between morphological groups by using one-way analysis of variance (ANOVA). For characters determined to be significant, we again tested each pair of morphological groups through Tukey’s pairwise test and used letters to identify groups that differed significantly. All statistical analyses were performed using PAST statistical software v.4 (Hammer et al. 2001).

**Table 2.** The abbreviation and meaning of morphological characters measured in this study.

Abbreviation	Meaning
Leaf	
LL	Leaf length
BL/BWL	The ratio of blade length to the length from the blade base to the most width part of the blade
BL/BW	The ratio of blade length to the most width of the blade
LL/BL	The ratio of leaf length to blade length
LL/PL	The ratio of leaf length to petiole length
Flower	
LT	Lobe thickness
SCL	The curve length of the stigma surface
LBST	The distance from the lobe base to the stigma apex
SDA	Stigma angle
SL	Stigma height
SW	Stigma width
LBW	Lobe base width
LBL	Lobe length
PTL2/PTBW	The ratio of the perianth tube length to the width of the perianth tube base
PTW1/PTBW	The ratio of the perianth tube width on the stamen-attached position to the width of the perianth tube base
PTW2/PTBW	The ratio of the perianth tube width on the stigma apex to the width of the perianth tube base
PTW3/PTBW	The ratio of the perianth tube width on the lobe base to the width of the perianth tube base
PTL1/PH	The ratio of the perianth tube length (exclude lobe) to pistil height
PTL1/SH	The ratio of the perianth tube length (exclude lobe) to the height of the stamen attached on the perianth tube (stamen height)
PTL1/LBL	The ratio of the perianth tube length (exclude lobe) to lobe length
PTL1/PTL2	The ratio of the perianth tube length (exclude lobe) to the perianth tube length
PH/SH	The ratio of pistil height to the height of the stamen attached on the perianth tube (stamen height)

**Table 3.** Leaf and floral quantitative characters of *Aspidistra* in Taiwan measured for the samples of the five *a priori* groups.

Characters		AA (N = 46) (mean ± stand. dev.)	ADE (N = 13) (mean ± stand. dev.)	ADS (N = 14) (mean ± stand. dev.)	AL (N = 2) (mean ± stand. dev.)	AM (N = 22) (mean ± stand. dev.)
Leaf	LL	0.745 ± 0.725 a	–0.100 ± 0.805 b	–1.048 ± 0.447 c	–1.742 c	–0.642 ± 0.574 bc
	BL/BWL	–0.585 ± 0.724 b	0.397 ± 0.879 a	0.644 ± 0.979 a	1.184 a	0.566 ± 0.901 a
	BL/BW	0.587 ± 0.428 a	0.017 ± 0.771 b	–1.881 ± 0.650 c	–0.587 b	–0.096 ± 0.614 b
	LL/BL	–0.182 ± 0.810 bc	0.393 ± 0.959 ab	–0.421 ± 0.659 bc	1.613 c	0.641 ± 1.194 a
	LL/PL	0.151 ± 1.019 b	–0.436 ± 0.484 bc	0.168 ± 0.882 bc	2.935 a	–0.496 ± 0.697 c
Flower	LT	0.237 ± 0.833 b	1.318 ± 0.935 a	–0.905 ± 0.561 c	–1.104 ± 0.386 bc	–0.600 ± 0.471 c
	SCL	0.770 ± 0.743 a	0.143 ± 0.385 b	–1.269 ± 0.365 c	–0.986 ± 0.114 bc	–0.815 ± 0.345 c
	LBST	0.569 ± 1.009 a	–0.056 ± 0.676 ab	–0.948 ± 0.377 cd	–2.148 ± 0.473 d	–0.215 ± 0.295 bc
	SDA	–0.898 ± 0.386 c	1.242 ± 0.296 a	0.552 ± 0.899 b	0.875 ± 1.428 ab	0.625 ± 0.447 b
	SL	0.842 ± 0.753 a	–0.749 ± 0.397 b	–0.727 ± 0.303 b	–0.364 ± 0.068 ab	–0.837 ± 0.533 b
	SW	–0.540 ± 0.936 c	1.470 ± 0.664 a	–0.287 ± 0.515 bc	0.127 ± 0.008 abc	0.369 ± 0.308 b
	LBW	0.184 ± 0.883 a	0.444 ± 1.004 a	–1.279 ± 0.679 b	1.670 ± 0.999 a	0.023 ± 0.720 a
	PTL2/PTBW	0.337 ± 0.989 a	0.484 ± 0.810 a	–0.132 ± 0.390 ab	–2.489 ± 0.313 c	–0.600 ± 0.769 b
	PTW1/PTBW	0.512 ± 1.066 a	–1.051 ± 0.377 b	–0.190 ± 0.877 ab	–0.402 ± 0.276 ab	–0.333 ± 0.461 b
	PTW2/PTBW	–0.173 ± 1.228	–0.283 ± 0.550	0.361 ± 0.889	0.244 ± 0.238	0.297 ± 0.650
	PTW3/PTBW	–0.234 ± 1.124	0.233 ± 0.831	0.057 ± 1.104	1.164 ± 0.156	0.228 ± 0.590
	PTL1/PH	–0.152 ± 0.739 bc	0.415 ± 0.996 ab	–0.531 ± 0.635 c	–2.376 ± 0.566 d	0.776 ± 0.951 a
	PTL1/SH	–0.364 ± 0.686 c	0.324 ± 0.667 ab	1.001 ± 1.232 a	–2.153 ± 0.040 d	–0.102 ± 0.559 bc
	PTL1/LBL	0.001 ± 0.863 b	–0.599 ± 0.786 bc	–0.128 ± 0.821 abc	–0.184 ± 0.177 c	0.693 ± 1.058 a
	PTL1/PTL2	0.504 ± 0.958 a	−0.602 ± 0.594 b	–0.622 ± 0.729 b	–1.322 ± 0.456 b	–0.020 ± 0.704 ab
	PH/SH	–0.211 ± 0.581 b	–0.091 ± 0.689 b	1.014 ± 1.038 a	0.016 ± 0.630 ab	–0.544 ± 0.477 b

The letters after the numbers were used to identify groups with significantly differences.

### ﻿DNA extraction and genotyping

DNA was extracted from 15 to 25 mg of leaf material (stored in silica gel) for 128 samples by using the Viogene Plant Genomic DNA Extraction Miniprep System (Viogene-Biotek, Taiwan). On the basis of the study conducted by Lu et al. (2020), five populations without morphological data, namely H, HWS, NHL, DJL, and DLJ, were temporally classified into different *a priori* groups (Table 1). Nine microsatellite markers were amplified using the method of Huang et al. (2014). Genotyping was performed in a 48-capillary sequencer (Labnet MultiGene Gradient, Labnet International Inc., USA) by using 1.5 μL of DNA, 1000 μL of HiDi formamide (Thermo Fisher Scientific, Taiwan), and 8.3 μL of GeneScan-500 LIZ Size Standard (Applied Biosystems, Warrington, U.K.). To identify alleles (sizes of amplified PCR products), the resulting chromatograms were interpreted using Peak Scanner software v.1.0 (Applied Biosystems, Foster City, CA).

### ﻿Identification of genetic groups

Genetic clusters were identified using the Bayesian clustering algorithm implemented in Structure v.2.3.4 (Pritchard et al. 2000) without *a priori* grouping. Ten independent runs were performed for each value of K ranging from 1 to 10 under a model assuming admixture and correlated allele frequencies (Falush et al. 2007). Each run comprised a burn-in period of 100,000 replications, followed by a run length of 1,000,000 Markov Chain Monte Carlo iterations. The results of replicated runs for each value of K from 1 to 10 were combined using Structure Harvester v.0.9.94 (Earl and von Holdt 2012). The optimal value of K was determined by calculating log-likelihood values and by using the ΔK method developed by Evanno et al. (2005). To visualize cluster assignments, the outputs of replicated runs were combined using CLUMPP v.1.1.2 (Jakobsson and Rosenberg 2007).

### ﻿Genetic diversity and genetic differentiation between genetic groups

We used Arlequin v3.5.2 (Excoffier and Lischer 2010) to analyze molecular variance (AMOVA) and estimate the genetic diversity of each genetic group on the basis of the number of polymorphic loci as well as observed and expected heterozygosities (*H*_O_ and *H*_E_) and the inbreeding coefficient (*F*). Differences in genetic diversity between genetic groups were characterized by computing the pairwise *F*_ST_.

## ﻿Results

### ﻿Definition of *a priori* groups

We identified five *a priori* groups on the basis of their morphological and geographical data. The names of these *a priori* groups were derived from one of the four recognized taxon names after matching with one (Table 1). (1) A total of 46 samples obtained from the higher mountain altitude of central to southern Taiwan were called “AA” because they corresponded to the species *A.attenuata*. (2) A total of 22 samples from central Taiwan (Defulan Trail and Shaolai Trail, Taichung City) were called “AM” because they corresponded to the species *A.mushaensis*. (3) A total of 14 samples from the Hengchun Peninsula were called “ADS” because they corresponded to the species *A.daibuensissensu*Lu et al. (2020). (4) A total of 13 samples, which had distinctly thick perianth lobes, corresponded to the taxon *A.daibuensissensu*Wang (2004) and Chao (2016) and were called “ADE.” (5) Finally, 2 samples from Huisun Experimental Forest Station, Nantou County, which had elongated stamen connectives, corresponded to the taxon *A.longiconnectiva* and were called “AL.”

### ﻿Morphological differentiation between *a priori* groups

**Figure 3.**
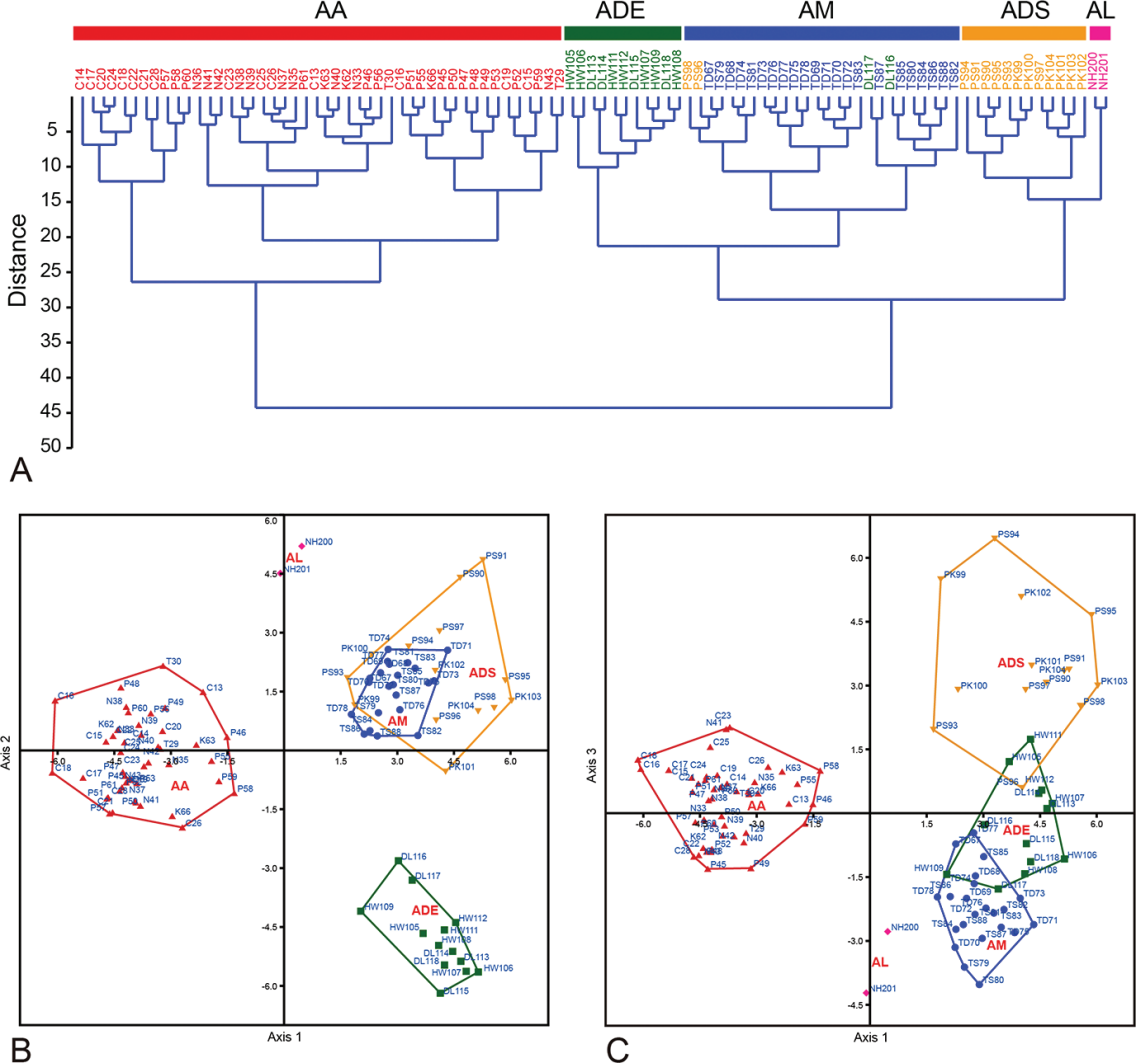
Results of morphometric analyses **A** dendrogram of clustering analysis **B, C** scatterplot of discriminant analysis **B** scatterplot by Axis 1× Axis 2 **C** scatterplot by Axis 1× Axis 3.

Fig. 3A presents the results of the CA based on the Ward’s method that was performed to evaluate the 5 leaf characters and 16 floral characters for the 97 specimens. The morphological clustering groups almost fitted *a priori* groups, except for some mismatched samples. In the CA dendrogram, the samples were first divided into two groups: the first group consisted of samples from the AA group, and the second group consisted of samples from the ADE, ADS, AL, and AM groups. The second group was divided into four subgroups, which corresponded to the four *a priori* groups. However, except for the AL group, the other three groups included some mismatched samples, such as two ADE samples, and two ADS samples were assigned to the AM group (Fig. 3A). Axes 1, 2, and 3 of DA together accounted for 94.96% of the total variance. Axis 1 (59.81% of the relative contribution) was mainly determined on the basis of the ratio of the pistil height to the stamen height (PH/SH) and the distance from the perianth tube lobe base to the stigma apex (LBST). Axis 2 (20.9% of the relative contribution) was mainly determined on the basis of the distance from the perianth tube lobe base to the stigma apex (LBST), the ratio of the perianth tube length to the width of the perianth tube base (PTL2/PTBW), and the curve length of the stigma surface (SCL). Axis 3 (14.25% of relative contribution) was determined on the basis of the ratio of the blade length to the blade width (BL/BW), PH/SH, and the ratio of the leaf length to the blade length (LL/BL). In DA scatterplots, the AA and AL groups were unambiguously separated along axis 1, whereas the other groups were less pronounced (Fig. 3B, C). The ADE group was separated from the AM and ADS groups along axis 2, but the AM and ADS groups overlapped in the axis 1–axis 2 scatterplot (Fig. 3B). In the axis 1–axis 3 scatterplot, the ADS group could be distinguished from the AM group along axis 3, whereas the ADE group was located between these two groups (Fig. 3C).

A comparison of paired means by using one-way ANOVA revealed that the quantitative variables significantly differed among the five *a priori* groups, except for PTW2/PTBW and PTW3/PTBW (Table 3). The results of Tukey’s pairwise test for each pair of morphological groups were as follows. The AA group significantly differed from the ADE, ADS, AL, and AM groups in terms of three of the five leaf characters (LL, BL/BWL, and BL/BW) and the stigma disk angle (SDA). The AL group significantly differed from all the other groups in terms of floral characters (PTL2/PTBW and ratio of the perianth tube length [excluding the lobe] to the pistil height [PTL1/PH], and PTL1/SH). The leaf and floral characters differed among the ADE, ADS, and AM groups. In terms of leaf characters, leaf length (LL) significantly differed between the ADE group and the other two groups, BL/BW significantly differed between the ADS group and the other two groups, and the ratio of the leaf length to the petiole length (LL/PL) significantly differed between the AM and the other two groups. In terms of floral characters, the ADE had significantly thicker perianth lobes. In addition, the SCL, SDA, and stigma width significantly differed between the ADE group and the other two groups. Compared with the other two groups, the ADS group had a significantly smaller lobe base width, a smaller PTL1/PH ratio, and a PH/SH ratio.

### ﻿Identification of genetic groups

**Figure 4.**
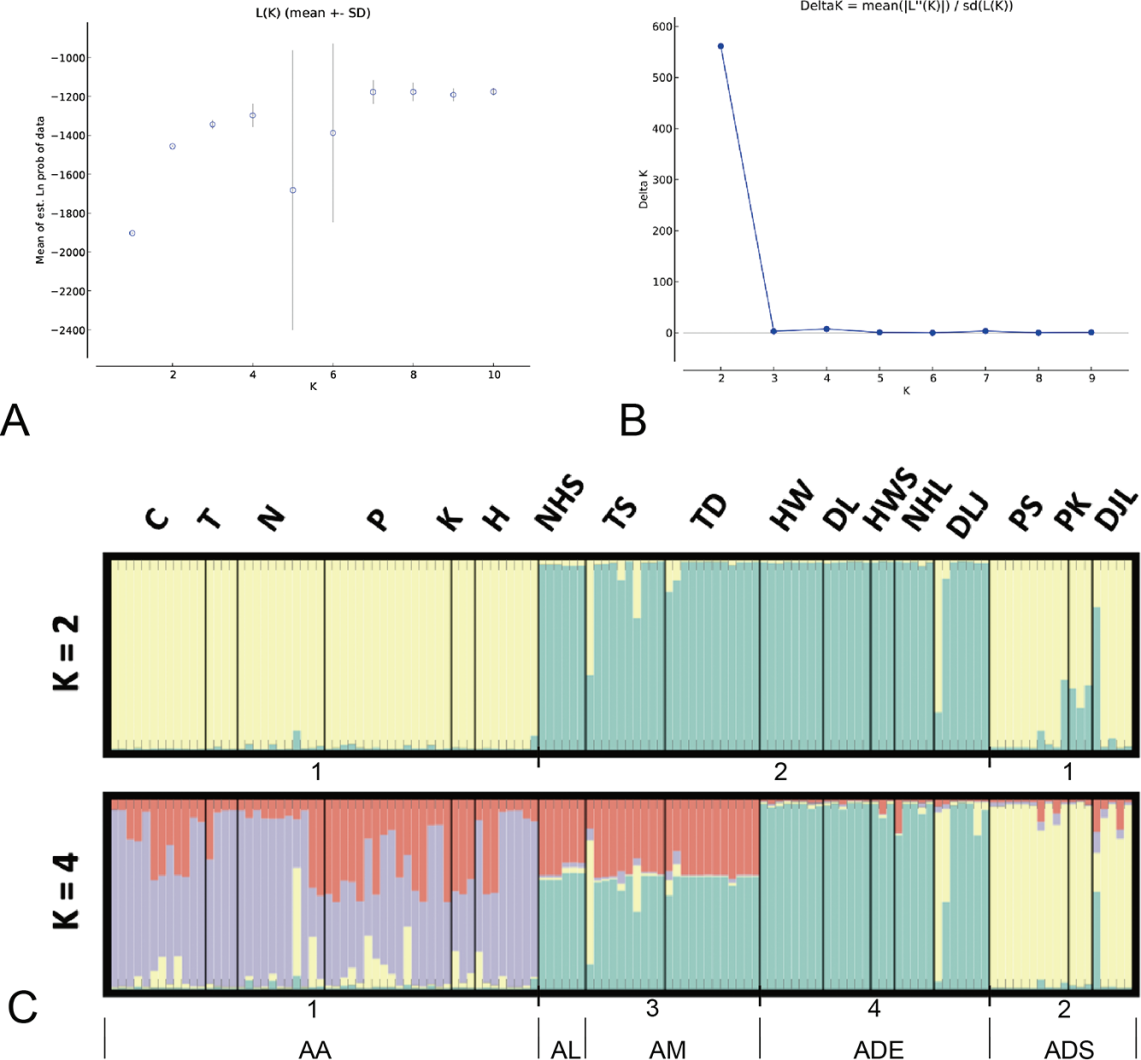
Results of genetic grouping **A, B** variation in the likelihood of data, L(K) (**A**) and of Delta K (**B**) as a function of the number of genetic groups (K) identified in 128 *Aspidistra* samples by using the software STRUCTURE **C** histogram of the genetic assignment of 128 *Aspidistra* individuals at K = 2 and K = 4. Individuals were ordered along the horizontal axis in accordance with their *в* group. Vertical bars indicate how the genome of each individual is partitioned into four clusters.

The results of Bayesian structuring analysis performed using nine nuclear microsatellites in Structure software (Pritchard et al. 2000) revealed that the likelihood of data substantially increased with the number of imposed clusters K until K reached a value of 2 and a plateau was reached for a larger K value (Fig. 4A). Application of the delta K method developed by Evanno et al. (2005) demonstrated that the K value of 2 was the most likely number of clusters (Fig. 4B). We found that genetic cluster 1 matched the AA and ADS groups, and that genetic cluster 2 matched the AM, ADE, and AL groups. However, when investigating clustering solutions obtained at higher K values, a good match between the genetic clusters and *a priori* groups was observed at the K value of 4 (Fig. 4C). Cluster 1 matched the AA group, cluster 2 matched the AM and AL groups, cluster 3 matched the ADS group, and cluster 4 matched the ADE group. Therefore, we used the K value of 4 to analyze differences in genetic diversity between different genetic groups.

Furthermore, the genetic clusters indicated that the five populations without morphological data, namely H, HWS, NHL, DJL and DLJ, were temporally classified into different *a priori* groups according to Lu et al. (2020) have slightly different results. The DJL population was formerly classified to the ADS group, but now was allocated to the ADE group.

### ﻿Genetic diversity and genetic differentiation between genetic groups

The genetic diversity of the genetic groups was substantial: the AL and AM groups displayed the highest diversity (*H*_E_ = 0.4403), whereas the ADE group exhibited the lowest diversity (*H*_E_ = 0.2842). The AA and ADS groups displayed intermediate diversity (*H*_E_ ranged from 0.3428 to 0.3951; Table 4A). The *H*_O_ of each genetic group was smaller than its *H*_E_.

**Table 4.** Genetic diversity (A) and differentiation (B) parameters for the four genetic groups of the genus *Aspidistra* in Taiwan detected using nine microsatellite markers.

**A. Genetic diversity parameters**					
	**Sample size**	**No. of polymorphic loci**	***H_O_***	***H_E_***	**F**
AA	54	8	0.2757 (±0.20422)	0.3428 (±0.24237)	0.1972*
ADE	29	7	0.1801 (±0.31215)	0.2842 (±0.25198)	0.3705*
ADS	18	7	0.3395 (±0.28252)	0.3951 (±0.27706)	0.1442*
AL+AM	28	9	0.3175 (±0.22335)	0.4403 (±0.22806)	0.2826*
**B. Genetic differentiation parameters (pairwise *Fst*)**					
	**AA**	**ADE**	**ADS**	**AL+AM**	
AA	0				
ADE	0.4939*	0			
ADS	0.2450*	0.3774*	0		
AL+AM	0.4105*	0.2710*	0.3761*	0	

*p < 0.05; *N*_A_: mean number of alleles observed per locus; *H*_E_: expected heterozygosity (unbiased estimator); *H*_O_: observed heterozygosity; *F*: inbreeding coefficient. *p < 0.01.

The inbreeding coefficient is a key parameter for understanding the number of matings between related individuals in a population. If no heterozygotes are present in a population, the inbreeding coefficient is 1.0. When the heterozygote frequency is equal to the Hardy–Weinberg expected value, the inbreeding coefficient is 0. Our results revealed that the inbreeding coefficients of the four genetic groups were significantly larger than 0, ranging from 0.1442 to 0.3705. This finding indicated that the degree of inbreeding varied within each group.

Pairwise *F*_ST_ is considered a satisfactory index to explain genetic differences among populations. Index values of 0–0.05, 0.05–0.15, 0.15–0.25, and >0.25 indicate nearly no, moderate, high, and significant genetic differences between populations, respectively (Wright 1978; Hartl and Clark 1997; Balloux and Lugon-Moulin 2002). Our results revealed that the pairwise *F*_ST_ between the genetic groups ranged from 0.2450 to 0.4939 (Table 4B). The pairwise *F*_ST_ of five of the six group pairs was >0.25. This result indicated the presence of significant genetic differences among the groups.

We determined differences in genetic diversity among the five *a priori* groups. The results were similar to the aforementioned results (Table 5). Except for the AL group, the *H*_O_ of the other four groups were all smaller than the *H*_E_, and the inbreeding coefficient values were all greater than 0, ranging from 0.1442 to 0.3705. The pairwise *F*_ST_ of almost all the pairs was >0.25.

**Table 5.** Genetic diversity (A) and differentiation (B) parameters for the five *a priori* groups of the genus *Aspidistra* in Taiwan detected using nine microsatellite markers.

**A. Genetic diversity parameters**					
	**Sample size**	**No. of polymorphic loci**	***H_O_***	***H_E_***	***F***
AA	54	8	0.2757 (±0.20422)	0.34281 (±0.24237)	0.1972*
ADE	29	7	0.1801 (±0.31215)	0.2842 (±0.25198)	0.3705*
ADS	18	7	0.3395 (±0.28252)	0.3951 (±0.27706)	0.1442*
AL	6	5	0.3333 (±0.43301)	0.3030 (±0.29545)	-0.1111
AM	22	9	0.3131 (±0.27125)	0.4091 (±0.18091)	0.2388*
**B. Genetic differentiation parameters (pairwise *Fst*)**					
	**AA**	**ADE**	**ADS**	**AL**	**AM**
AA	0				
ADE	0.49393*	0			
ADS	0.24497*	0.37740*	0		
AL	0.45724*	0.41715*	0.44917*	0	
AM	0.44411*	0.30664*	0.40586*	0.28430*	0

*p < 0.05; *H*_E_: expected heterozygosity (unbiased estimator); *H*_O_: observed heterozygosity; *F*: inbreeding coefficient. *p < 0.01.

## ﻿Discussion

### ﻿Comparison with morphological and *a priori* groups

The CA and DA of leaf and floral characters revealed that the five *a priori* groups were well-supported by morphological groups (Fig. 3). Both the CA dendrograms and DA scatterplots demonstrated that the AA group differed from the AL group. The AA group corresponded to *A.attenuata*, and the AL group corresponded to *A.longiconnectiva*. With its longer leaves and concave stigma, *A.attenuata* exhibits clear morphological differentiation. Several authors have recognized all these characters to differentiate species (Hayata 1912; Liu and Ying 1978; Yang et al. 2001; Wang 2004; Chao 2016; Lu et al. 2020). *A.longiconnectiva* is characterized by deep perianth lobes, relatively broad and shallow perianth tubes, and elongated stamen connectives (Lu et al. 2020). The ADE, ADS, and AM groups corresponded to the eastern population of *A.daibuensis*, the southern and southeastern populations of *A.daibuensis*, and *A.mushaensis*, respectively. However, some mismatched samples were noted within these three groups. These mismatched samples may be attributed to the ambiguous morphological characters between the two species. This result is consistent with those of previous taxonomic studies. Wang (2004) and Chao (2016) have considered that the southern and southeastern populations of *A.daibuensis* correspond to *A.mushaensis*, and that the eastern population of *A.daibuensis* corresponds to *A.daibuensis*. However, Lu et al. (2020) believed that *A.mushaensis* exists only in central Taiwan, and that the southern and southeastern populations of *A.daibuensis* correspond to *A.daibuensis* and not *A.mushaensis*. Whether the ADS group corresponds to *A.daibuensis* or *A.mushaensis* remains unclear. However, our morphometric analyses revealed that the ADS group differed from the AM and ADE groups in terms of a wider blade width, a smaller lobe base width, more stigma present near the perianth opening, and the stamen attached on the perianth tube base or near the base. Thus, we suggest that the ADS group should not be classified as the AM or ADE group.

### ﻿Comparison with genetic groups and *a priori* groups

The results of Bayesian structural analysis revealed that a K value of 2 was the most favorable for genetic clustering. Genetic cluster 1 consisted of the AA and ADS group, and genetic cluster 2 consisted of the ADE, AL, and AM groups. However, this genetic division was inconsistent with the morphological division based on CA, wherein in the CA dendrogram, the samples were divided into the AA group and the other groups (ADE, ADS, AL, and AM). Although two clusters were obtained in our dataset based on ∆K, we determined that a K value of 4 led to more favorable matching with the *a priori* groups. Demographic, environmental, and historical processes are multifaceted and complex and have led to different organization levels in the genetic structure of species. Therefore, Meirmans (2015) suggested that different K values may reflect different demographic processes, and that a biologically interpretable pattern obtained from a suboptimal K value is better than a completely unrealistic pattern obtained from the optimal K value. Thus, we used the K value of 4 to determine the relationship between genetic and morphological groups. Although genetic clusters almost supported our morphological groups, AL clustered with AM to form a genetic group. This result can be attributable to the small sample size and incomplete lineage sorting. First, the AL group corresponded to *A.longiconnectiva*, which is a rare species in Taiwan, and we only sampled six individuals from a locality. The Structure software usually fails to identify genetic groups represented by few individuals, even when one group is well-represented (Porras-Hurtado et al. 2013; Wang 2017). Second, AL and AM are morphologically similar (Lu et al. 2020) and are sympatrically distributed. We believe that AL and AM are closely related and may have diverged recently. Genetic data also indicated that they were closely related. Thus, the lineage sorting of these two species is incomplete.

### ﻿Genetic diversity and genetic differentiation between genetic groups

The inbreeding coefficient indicates the effect of inbreeding on homozygosity by quantifying the deviation in observed genotypic frequencies from those expected under the Hardy–Weinberg equilibrium (Charlesworth 2003). Inbreeding, null alleles, and undetected genetic structure increase observed homozygosity and the inbreeding coefficient, whereas outbreeding, mutation, and inbreeding depression tend to reduce the inbreeding coefficient (Stoeckel et al. 2006; Waples 2018). Our results revealed that the inbreeding coefficients of all the genetic clusters were significantly greater than 0. Therefore, each genetic group had a high possibility of inbreeding. Inbreeding promotes population fragmentation and provides a selective advantage at the population level, resulting in structured populations, which contribute to speciation (Joly 2010; Olsen et al. 2020).

In our genetic differentiation analysis, we observed significantly high pairwise *F*_ST_ values, ranging from 0.2450 to 0.4939. Among the pairwise *F*_ST_ values of the six genetic group pairs, the AA with ADE pair and the AA with AL+AM pair exhibited high genetic differentiation (0.4939 and 0.4105, respectively), whereas the pairwise *F*_ST_ values of the AA with ADS pair was slightly smaller (0.2450) but still indicated high genetic differentiation (Wright 1978; Hartl and Clark 1997; Balloux and Lugon-Moulin 2002). The remaining genetic group pairs, namely ADE with ADS, ADE with AL+AM, and ADS with AL+AM, had significantly high pairwise *F*_ST_ values (0.3774, 0.2710, and 0.3761, respectively). We believe that these three groups have high genetic differentiation. This result also supports our morphometric analysis finding that ADE, ADS, and AM are distinct entities.

### ﻿Integration of morphometric and genetic data

We performed an iterative analysis of morphometric and microsatellite markers to define, assess, and delineate species within the *Aspidistra* genus in Taiwan. The integration of the two analyses was almost congruent for the five *a priori* groups. In addition, the population genetic data indicated that frequent inbreeding and high genetic differentiation may shape the species diversity of *Aspidistra* in Taiwan. Therefore, we suggest that the five *a priori* groups should be regarded as five different taxa. Considering the morphological similarities between these five taxa, their geographical distribution as well as the preliminary ranking rule providing by Tillich and Averyanov (2018), we proposed the following taxonomic treatments to update the taxonomy of *Aspidistra* in Taiwan: AA corresponds to the species *A.attenuata*, which is distributed in the mountain range at higher altitudes in the central and southern Taiwan; AM corresponded to the species *A.mushaensis*, which is only distributed in central Taiwan; AL corresponded to the species *A.longiconnectiva*, because it is genetically similar to *A.mushaensis*, we reduced it to the variety level; ADE corresponded to the species *A.daibuensis*, which has restricted distribution in southeastern Taiwan; ADS morphologically resembles ADE, but they differ in their genetic data. Therefore, we considered regarding it as a new variety of *A.daibuensis*.

## ﻿Conclusion

Our study demonstrated that combining morphological and population genetic data is effective for the discovery of new *Aspidistra* taxa. By using this approach, we confirmed five *Aspidistra* taxa in Taiwan, namely *A.attenuata* Hayata; A.daibuensisHayatavar.daibuensis; A.mushaensisvar.longiconnectiva (C.T.Lu, K.C.Chuang & J.C.Wang) C.T.Lu & J.C.Wang; A.mushaensisHayatavar.mushaensis; and a new variety, A.daibuensisHayatavar.longkiauensis C.T.Lu, M.J.Yang & J.C.Wang var. nov. Combining morphometric and genetic studies can help us discover species diversity in this genus. The complete description of the new variety is provided in the taxonomic treatment below.

## ﻿Taxonomic treatment

### ﻿ Aspidistra daibuensis Hayata var. longkiauensis

Taxon classificationPlantaeAsparagalesAsparagaceae

C.T.Lu, M.J.Yang & J.C.Wang var. nov.

34F1273A-4851-5C21-AD9C-4A678A82E524

urn:lsid:ipni.org:names:77316103-1

**Figure 5.**
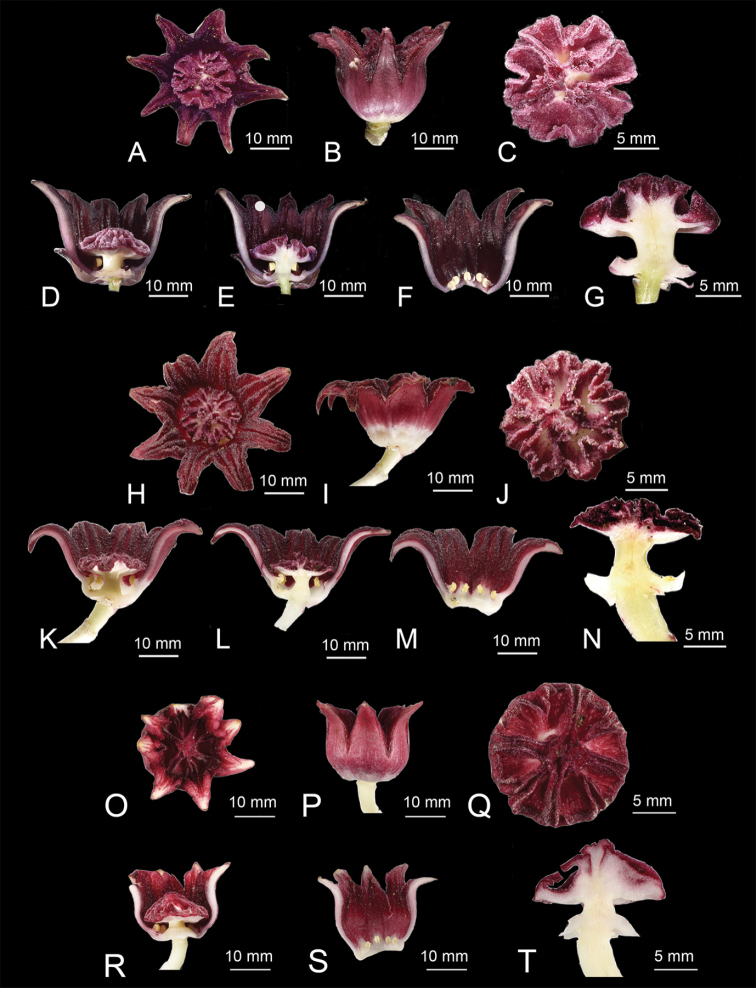
Floral comparison of Aspidistradaibuensisvar.longkiauensis C.T.Lu, M.J.Yang & J.C.Wang; A.mushaensisHayatavar.mushaensis, and A.daibuensisHayatavar.daibuensis**A–G**A.daibuensisvar.longkiauensis**H–N**A.mushaensisvar.mushaensis**O–T**A.daibuensisvar.daibuensis**A, H, O** front view of the flower **B, I, P** lateral view of the flower **C, J, Q** stigma surface **D, K, R** half dissection of the perianth tube, showing the pistil and stamens **E, L** half dissection of the perianth tube and stigma **F, M, S** half dissection of the perianth tube, showing the stamen **G, N, T** half dissection of the stigma.

**Figure 6.**
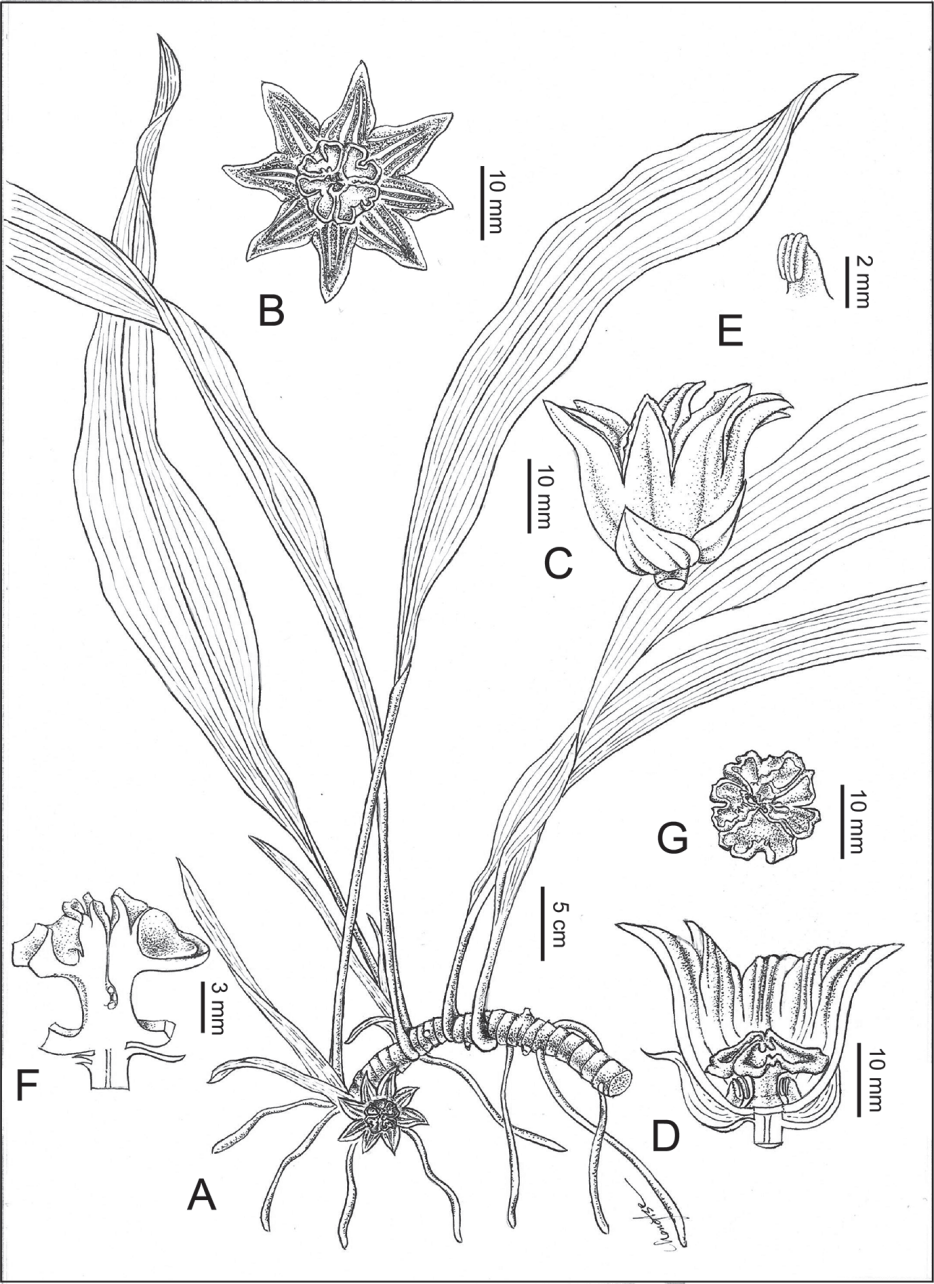
Illustration of Aspidistradaibuensisvar.longkiauensis C.T.Lu, M.J.Yang & J.C.Wang **A** habit **B** flower (front view) **C** flower (lateral view) **D** half dissection of perianth tube, showing pistil and stamens **E** stamen **F** half dissection of pistil.

Figs 5A–G , 6 Chinese name: 瑯嶠蜘蛛抱蛋

#### Diagnosis.

This new variety is similar to A.daibuensisHayatavar.daibuensis but can be distinguished by its shorter leaf length (72.24 ± 11.68 cm vs. 95.46 ± 21.04 cm), smaller ratio of the leaf blade length to width (3.73 ± 1.43 vs. 8.05 ± 1.73), thinner perianth lobe (1.28 ± 0.38 mm vs. 2.79 ± 0.64 mm), longer curve length of the stigma surface (10.98 ± 1.38 mm vs. 16.23 ± 1.45 mm), and larger ratio of the pistil height to stamen height (2.49 ± 0.59 vs. 2.03 ± 0.78). It also resembles A.mushaensisHayatavar.mushaensis but differs by its smaller ratio of the leaf blade length to width (3.73 ± 1.43 vs. 7.66 ± 1.35), narrower perianth lobe base (5.12 ± 1.12 mm vs. 7.27 ± 1.19 mm), and larger ratio of the pistil height to the stamen height (2.49 ± 0.59 vs. 1.59 ± 0.26).

#### Type.

Taiwan. Pintung County, Shuangliou national forest recreation area, Banyan trail, elev. 200–300 m, 12 Jun 2020, *M.J.Yang s.n.* (holotype: TAIF; isotype: TNU).

#### Description.

Perennial evergreen herb. Rhizome creeping, 1–1.2 cm in diameter. Internode 0.2–0.4 cm. Cataphylls 3 to 4. Leaves: coriaceous, solitary or rare 2 tufts leaves, blade dark green occasionally with yellow spots; leaf margin with white serrulate, parallel venation; petioles 9.6–36.6 cm long; blade oblanceolate, blade oblique, 40.5–61.8 cm long, 4.6–8.6 cm wide, acute at apex, . Flower solitary, bisexual, scape 0.3–4.0 cm long, 3–6 bracts, bract ovate; perianth urceolate to campanulate, fleshy, perianth tube purple, basal white, 13.5–20.5 mm long, 8.5–15.5 mm wide; perianth lobe 7–9, sometimes yellow at apex, ovate triangular, 5–10 mm long, base 3.2–7.4 mm wide, 0.7–2.0 mm thickness, lobe center with two keels, lobe margin with two keels conspicuous. Stamens 7–9 as many as the perianth lobe, inserted at the perianth tube base or near base; anther oblong, 1.3–3.0 mm long; anther connective absent; filament short, 1–2 mm. Pistil 5–8 mm; stigma disk-like, 4 to 5 lobes, conical and lobe marginal concave, stigma nearly covering the perianth tube, 7.6–12.8 mm wide; style 0.5–1.5 mm long; ovary 1.2–1.7 mm with 4–5 locules. Fruit unknown.

#### Etymology.

The specific epithet “*longkiau*” means that the geographical distribution of this species is mainly distributed in Hengchun Peninsula, Pingtung County, Taiwan. The area was known as “Longkiau” in early records.

#### Phenology.

Flowering from February to June.

#### Distribution and habitat.

Aspidistradaibuensisvar.longkiauensis is geographically confined to the Hengchun Peninsula, the most southern part of Taiwan and the southeastern part of Taiwan. The population typically grows on slopes under the canopies of primary or secondary forests. It is distributed at an elevation of 200–500 m.

#### Conservation status.

Aspidistradaibuensisvar.longkiauensis is known from three populations in Hengchun Peninsula, Pingtung County, Taiwan. Based on the specimen records, the area of occupancy (AOO) is ca. 15 km^2^ by GeoCAT (Bachman et al. 2011). Following the criteria of IUCN (2019), this species is assessed as endangered (EN B2ab(ii)).

#### Notes.

This new variety is regarded as *A.daibuensis* (Chang and Hsu 1974) or *A.mushaensis* (Wang 2004; Chao 2016). We determined that this species does not have thick perianth lobes as A.daibuensisvar.daibuensis (Hayata 1912). In addition, A.daibuensisvar.longkiauensis differs from A.mushaensisvar.mushaensis in terms of the ratio of the blade length to the blade width, perianth lobe base width, the distance from the lobe base to the stigma apex, and the ratio of the pistil length to the stamen height (Table 6). The stamen of this new species is inserted on the perianth tube base or near the base instead of the low part of the perianth tube, as observed in the previous former two species. We considered it can be distinguished from A.daibuensisvar.daibuensis and *A.mushaensis* var. mushaensis. However, it is morphologically more similar to *A.daibuensis*, so we considered regard it as a variety of *A.daibuensis*.

**Table 6.** Morphological comparison of Aspidistradaibuensisvar.longkiauensis with A.daibuensisvar.daibuensis, A.mushaensisvar.longiconnectiva and A.mushaensisvar.mushaensis. *. Mean ± S.D. (min. value and max. value).

Taxa characters	A.daibuensisvar.longkiauensis	A.daibuensisvar.daibuensis	A.mushaensisvar.longiconnectiva	A.mushaensisvar.mushaensis
**Leaves**				
Leaf length (cm)	72.24 ± 11.68 (54.1–86.5)*	95.46 ± 21.04 (70.2–141.1)	54.1	82.87 ± 14.99 (60.1–106.1)
Leaf length/petiole length	3.40 ± 0.90 (2.4–4.0)	2.83 ± 0.51 (1.9–3.6)	1.19	2.72 ± 0.71 (1.7–3.6)
Blade length/blade width	3.73 ± 1.43 (1.4–6.4)	8.05 ± 1.73 (5.1–10.1)	6.58	7.66 ± 1.35 (6.1–10.1)
**Fowers**				
Perianth lobe thickness (mm)	1.28 ± 0.38 (0.7–2.0)	2.79 ± 0.64 (1.7–4.0)	1.14 ± 0.27 (1.0–1.3)	1.48 ± 0.34 (1.1–1.9)
Lobe base width (mm)	5.12 ± 1.12 (3.2–7.4)	7.95 ± 1.60 (5.8–9.3)	10.0 ± 1.66 (8.8–11.2)	7.27 ± 1.19 (5.5–8.7)
Curve length of the stigma surface (mm)	10.98 ± 1.38 (9.4–13.4)	16.23 ± 1.45 (13.7–17.5)	12.07 ± 0.43 (11.8–12.4)	12.70 ± 1.30 (10.4–15.7)
Stigma width (mm)	10.33 ± 1.79 (7.6–12.8)	16.43 ± 2.21 (12.7–21.4)	11.76 ± 0.03 (11.7–11.8)	12.59 ± 1.08 (10.2–14.6)
Ratio of the perianth tube length (excluding the lobe) to pistil height	1.71 ± 0.24 (1.3–2.2)	2.02 ± 0.41 (1.3–2.9)	0.91	2.19 ± 0.35 (1.6–3.1)
Ratio of perianth tube length (excluding the lobe) to stamen height	4.20 ± 0.86 (3.4–6.5)	3.88 ± 0.72 (2.7–5.9)	1.99 ± 0.03 (1.9–2.0)	3.43 ± 0.39 (2.7–4.7)
Ratio of perianth tube length to the width of the perianth tube base	1.52 ± 0.13 (1.4–1.8)	1.72 ± 0.27 (1.4–2.2)	0.73 ± 0.11 (0.7–0.8)	1.37 ± 0.26 (1.0–1.9)
Ratio of pistil height to stamen height	2.49 ± 0.59 (1.8–3.8)	2.03 ± 0.78 (1.3–2.5)	1.91 ± 0.35 (1.7–2.2)	1.59 ± 0.26 (1.0–2.1)

#### Additional specimen examined.

**Taiwan. Pintung County**: Kaoshifo, alt. 400 m, 14 Jun 1993, fl., *T.-T. Chen et al. 1393* (TAIF!); Mt. Kaoshifo, 27 Feb 2016, fl., *P.F. Lu 29262* (TAIF!); Mt. Kaoshifo, alt. 300–400 m, 16 April 2020, fl., *C.T. Lu 2662* (TNU!); Shoucha, alt. 470–500 m, 17 Feb 2017, fr., *S.W. Chung 12866* (TAIF!).

### ﻿Key to the species of *Aspidistra* in Taiwan

**Table:** 

1	Leaf blade length to leaf blade width > 10; stigma surface concave or flat; anthers inserted at one third of the perianth tube	***A.attenuata***
–	Leaf blade length to leaf blade width ≤ 10; stigma surface convex; anthers inserted in the low part of the perianth tube or near the perianth tube base	**2**
2	Leaf length to leaf blade length < 1.2; the perianth tube length to the width of the perianth tube base < 0.9; the perianth tube length (exclude lobe) to the height of the stamen attached on the perianth tube < 2.1	**A.mushaensisvar.longiconnectiva**
–	Leaf length to leaf blade length > 1.2; the perianth tube length to the width of the perianth tube base > 0.9; the perianth tube length (exclude lobe) to the height of the stamen attached on the perianth tube > 2.5	**3**
3	The perianth tube length (exclude lobe) to pistil heigh > 1.5	**A.mushaensisvar.mushaensis**
–	The perianth tube length (exclude lobe) to pistil heigh < 1.5	**4**
4	The curve length of the stigma surface > 13.5; lobe thickness up to 4 mm	**A.daibuensisvar.daibuensis**
–	The curve length of the stigma surface < 13.5; lobe thickness no more than 2 mm	**A.daibuensisvar.longkiauensis**

## Supplementary Material

XML Treatment for Aspidistra daibuensis Hayata var. longkiauensis
